# Supplementary material for: A New Strategy for Disc Cutter Wear Status Perception Using Vibration Detection and Machine Learning
Source: Sensors (Basel). 2022 Sep 4;22(17):6686. doi: 10.3390/s22176686 (PMC9459918; doi:10.3390/s22176686)
Supplement: Supplementary file 1 [file sensors-22-06686-s001.zip › sensors-1844263-supplementary.pdf]

A New Strategy for Disc Cutter Wear Status Perception Using Vibration Detection and Machine Learning

Xiaobo Pu [1,2], Lingxu Jia [1], Kedong Shang [1], Lei Chen [1], Tingting Yang [1,*], Liangwu Chen [2], Libin Gao [2] and Linmao Qian [1]

1 *Tribology Research Institute, State Key Laboratory of Traction Power, Southwest Jiaotong University, Chengdu 610031, China*

2 *China Railway Engineering Equipment Group Technical Service Co., Ltd., Zhengzhou 450000, China*

* *Correspondence: yangtingting@swjtu.edu.cn*

Table S1. Test bench specifications

Overall Dimensions (mm)	4939×1800×2347	Horizontal Guide Rail (mm)	3300(amount 3)
Rock Box Dimensions (mm)	1150×580×280	Disc Cutter Specifications (in.)	12 ~ 19
Horizontal Traction Speed (m/s)	0 ~ 1 (variable)	Screw Rod Specifications (mm)	Tr120×6-330
Total Weight (t)	approx. 10	Effective Stroke of Screw Rod (mm)	230

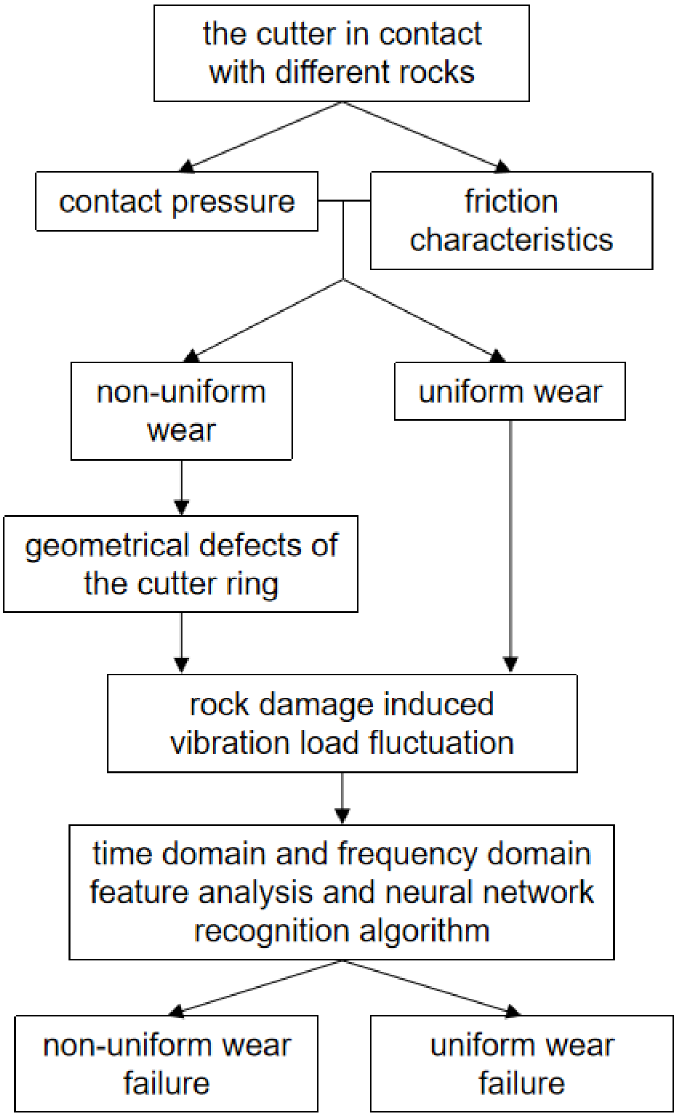

Figure S1. Overall logic of this research

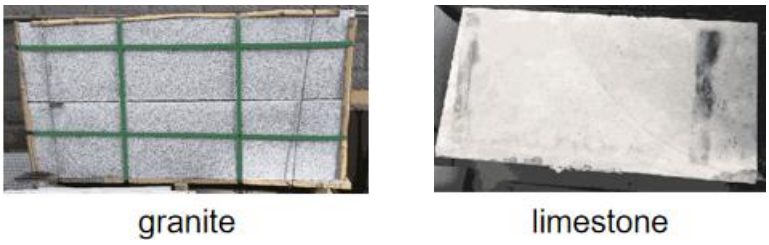

Figure S2. 2 types of rock samples

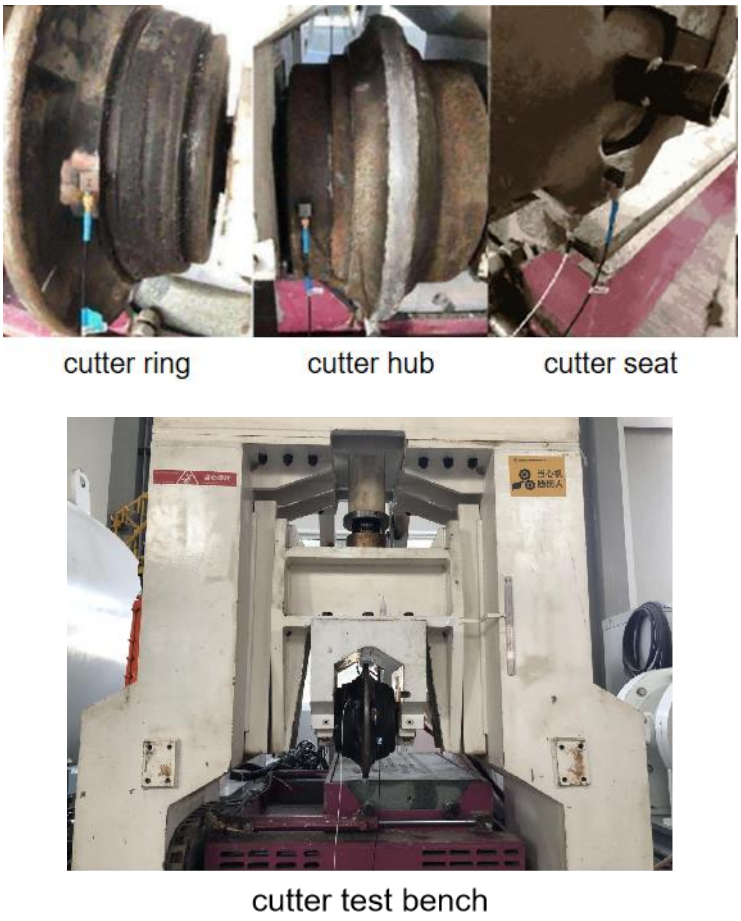

Figure S3. 3 locations of the sensor and test bench, physical representation

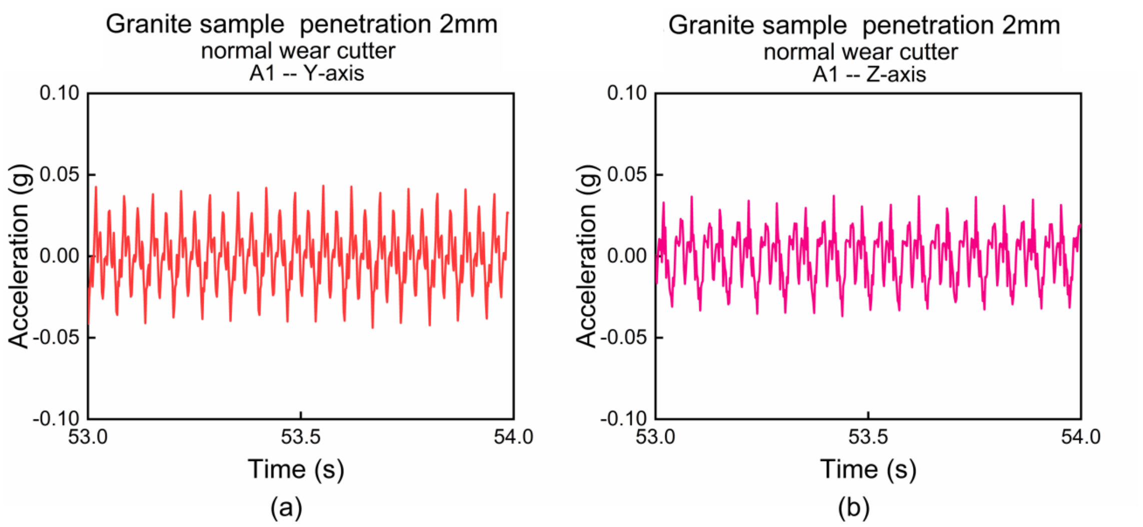

Figure S4. Granite sample, penetration 2mm, normal wear cutter, time domain waveforms: (**a**) periodic waveforms of Y-axis; (**b**) periodic waveforms of Z-axis

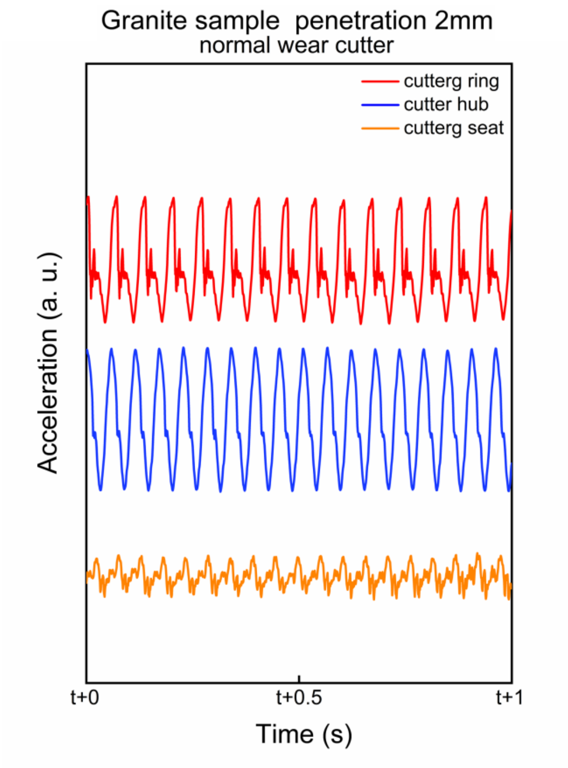

Figure S5. Granite sample, penetration 2mm, normal wear cutter, the detection results of the sensor at different positions

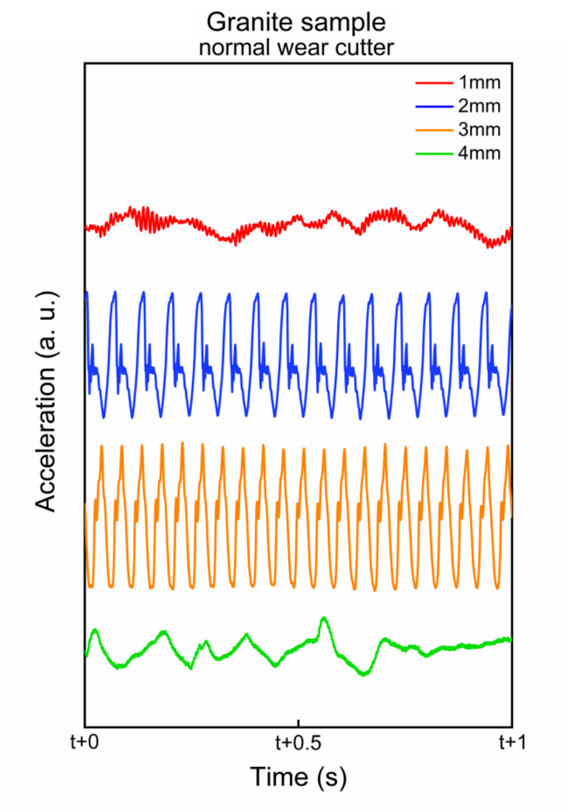

Figure S6. Granite sample, normal wear cutter, test results under different penetration conditions

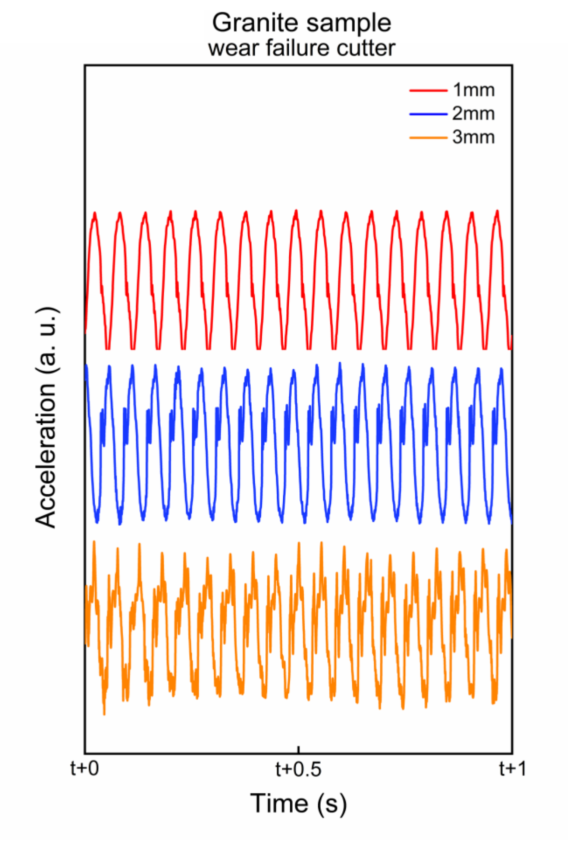

Figure S7. Granite sample, wear failure cutter, test results under different penetration conditions
